# Reaction temperature sensing (RTS)-based control for Li-ion battery safety

**DOI:** 10.1038/srep18237

**Published:** 2015-12-11

**Authors:** Guangsheng Zhang, Lei Cao, Shanhai Ge, Chao-Yang Wang, Christian E. Shaffer, Christopher D. Rahn

**Affiliations:** 1 Electrochemical Engine Center (ECEC), and Department of Mechanical & Nuclear Engineering, The Pennsylvania State University, University Park, PA 16802, USA; 2 EC Power, 341 Science Park Road, State College, PA 16803.

## Abstract

We report reaction temperature sensing (RTS)-based control to fundamentally enhance Li-ion battery safety. RTS placed at the electrochemical interface inside a Li-ion cell is shown to detect temperature rise much faster and more accurately than external measurement of cell surface temperature. We demonstrate, for the first time, that RTS-based control shuts down a dangerous short-circuit event 3 times earlier than surface temperature- based control and prevents cell overheating by 50 °C and the resultant cell damage.

Safety of large-scale Li-ion batteries has become a critical issue, propelled by ever-increasing applications in electric vehicles (EVs)^1^,^2^,^3^,^4^,^5^. Recent high-profile events involving electric cars and airplanes that employ Li-ion batteries as main or auxiliary power sources lend further urgency to improved Li-ion battery safety^6^,^7^. When safety is breached, Li-ion batteries may experience thermal runaway causing significant equipment or personnel damage^3^,^8^,^9^.

While Li-ion materials and chemistry have been well understood for over two decades, the engineering science of large Li-ion batteries remains elusive, as evident from the recent battery fire accident on Boeing Dreamliner 787. Early detection and protection technologies are essential elements of large Li-ion battery systems for vehicle, aircraft and grid energy storage applications^9^,^10^. Ability to measure the temperature precisely at the electrochemical reaction interface inside a cell provides sensitive monitoring of the health and safety states of the Li-ion cell^9^,^11^,^12^,^13^,^14^,^15^. The close correlation of the reaction temperature to battery internal state offers an excellent means for early detection of potential faults allowing timely intervention.

Some previous studies^16^,^17^,^18^,^19^,^20^ and our recent study^21^ on Li-ion battery internal temperature show that the internal temperature at the reaction area responds faster than surface temperature, especially when heat is generated rapidly such as during short circuit and overcharge^16^,^17^,^18^.We hypothesize that early detection and prevention of Li-ion battery safety failure can be realized through internal reaction temperature sensing (RTS) and a control algorithm based on RTS. Different from existing approaches that monitor voltage, current, or other sophisticated signal processing methods^10^,^22^,^23^,^24^,^25^,^26^,^27^,^28^ of individual cells or modules for early signs of abnormality, RTS directly monitors the temperature at the reaction surface, a critical parameter determining safety status of the Li-ion battery.

In this study, a novel RTS-based control strategy is developed to improve Li-ion battery safety. Different from previous studies^16^,^17^,^18^ in which internal temperature is just measured and compared with surface temperature during abuse testing, this study focuses more on using the faster response of internal reaction temperature for early detection and prevention of battery safety failure. This RTS-based control strategy can detect abnormal temperature rise inside Li-ion battery cells much faster and much more accurately than conventional surface temperature based control, thereby allowing for earlier intervention to prevent damage from safety breach. We demonstrate the effectiveness of this technique by fabricating Li-ion battery cells with internal reaction temperature sensors and testing them under a short-circuit condition, a common safety failure scenario for Li-ion batteries. By terminating the shorting when cell internal reaction temperature or surface temperature reaches a prescribed threshold, say 80 °C, we show that RTS-based control provides very effective early detection and prevention of Li-ion battery safety failures.

## Results

### Shorting test with RTS-based control

**Figure 1:**
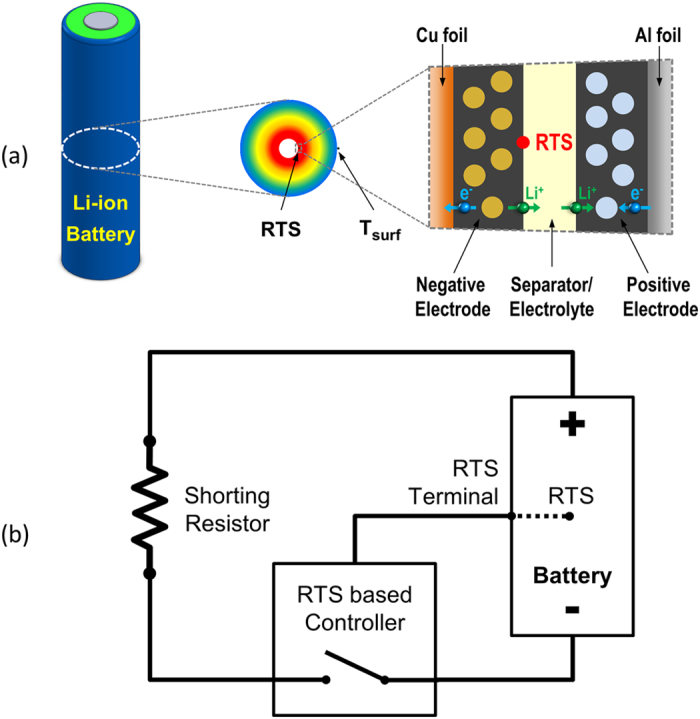
Schematics of reaction temperature sensing (RTS)-based control for Li-ion battery safety. (**a**) A cylindrical Li-ion cell with embedded RTS. (**b**) An experimental system that terminates shorting automatically when RTS input reaches threshold.

To demonstrate the effectiveness of RTS-based control for Li-ion battery safety, experimental Li-ion cell with embedded RTS sensor is fabricated in the Battery Manufacturing Lab at The Pennsylvania State University. Figure 1(a) shows the schematic of a cylindrical cell with an embedded RTS. A similar temperature sensor is also placed on the cell outer surface for comparison between RTS and external surface temperature (T_surf_). The experimental cell in this study has a nominal capacity of 1.6 Ah. It is fully charged and then short circuited using a specially designed experimental system that can terminate shorting automatically when RTS reaches threshold. A schematic of the experimental system is shown in Fig. 1(b). Details of fabricating RTS cells and developing the experimental system are described in the Methods section.

**Figure 2:**
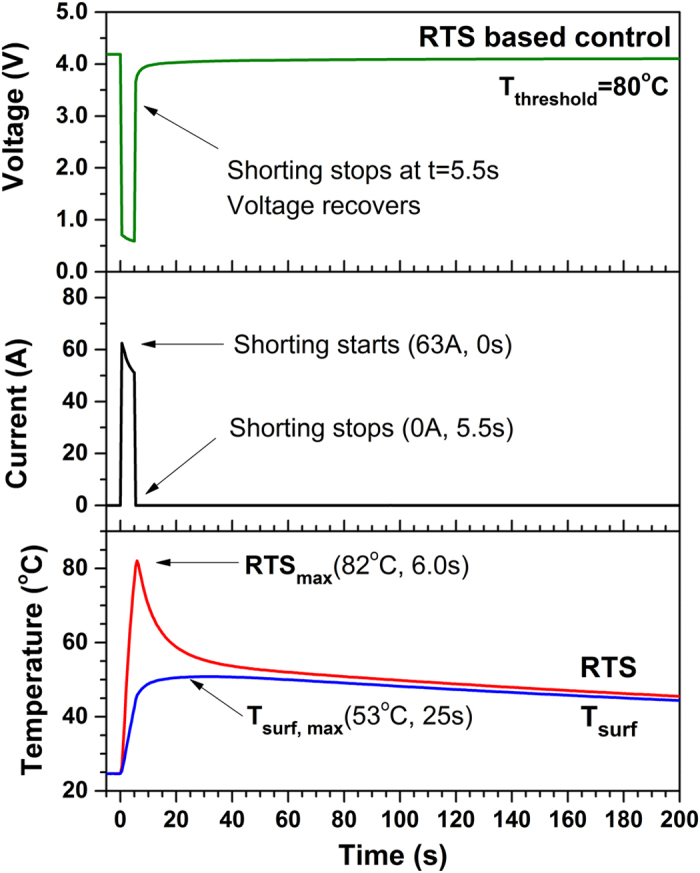
Variation of cell voltage, current, reaction temperature (RTS) and surface temperature (T_surf_) of experimental Li-ion cell during shorting test with RTS-based control (T_threshhold_=80 °C). At 5.5 s the reaction temperature reaches 80 °C and the shorting is terminated successfully. In comparison, the surface temperature is only 50 °C at shorting termination.

Figure 2 shows the variation of cell voltage, current, reaction temperature (RTS) and surface temperature (T_surf_) of the cell during shorting test with RTS-based control. The threshold temperature is 80 °C. This threshold is chosen because decomposition of solid electrolyte interface (SEI) and electrolyte can occur at around 80 °C^29^,^30^. It can be seen that as shorting begins, the voltage drops abruptly to around 0.7 V and the current reaches as high as 63 A, approximately 40 times that during 1C discharge. Such voltage and current behaviors are typical for Li-ion battery cells during short circuit^31^. Very low cell voltage and very high current suggests high rate of heat generation. While both reaction temperature and surface temperature increase dramatically, reaction temperature increases much more rapidly than surface temperature. At 5.5 s the reaction temperature reaches 80 °C and the shorting is terminated successfully by RTS-based controller. Then the reaction temperature reaches maximum value immediately and begins decreasing. In comparison, the surface temperature is only 50 °C at shorting termination and keeps increasing due to heat transfer from the cell interior. It reaches maximum value of 53 °C at 25 s, much lower and much later than the reaction temperature. Comparison with reaction temperature shows that surface temperature responds much more slowly and fails to detect the highest temperature a Li-ion battery cell could experience during abnormal operation. This successful termination of short circuit suggests that RTS-based control is effective in early detection and prevention of safety failure of Li-ion battery cells.

### Shorting test with surface temperature- based control

**Figure 3:**
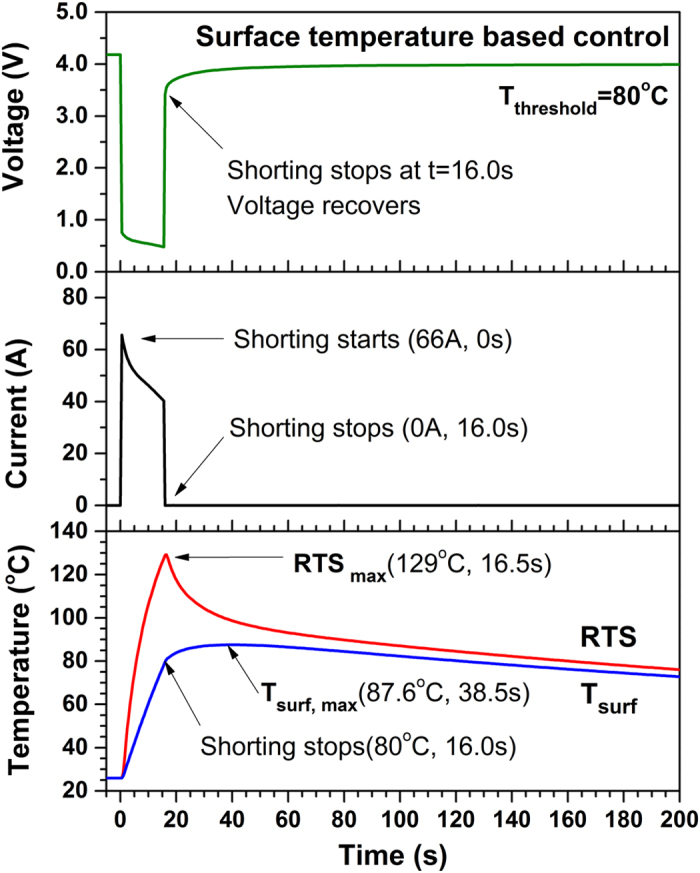
Variation of cell voltage, current, reaction temperature (RTS) and surface temperature (T_surf_) of experimental Li-ion cell during shorting test with surface temperature- based control (T_threshhold_=80 °C). The shorting is terminated when surface temperature reaches 80 °C, but it lasts 16.0 s, ~3 times of that with RTS-based control. The maximum reaction temperature reaches ~50 °C higher than the threshold temperature.

To further demonstrate the advantage of RTS-based control over surface temperature- based control, the cell is short circuited one more time with termination of shorting based on the surface temperature sensing. The threshold temperature is still kept at 80 °C. Figure 3 shows variation of cell voltage, current, reaction temperature and surface temperature (T_surf_) of the cell during test with surface temperature- based control. It can be seen that cell voltage, current, reaction temperature and surface temperature behave similarly to those in the previous case as shorting starts. The shorting is terminated successfully when the surface temperature reaches 80 °C, but lasts 16 s before termination. This is 3 times longer than demonstrated with RTS-based control. Moreover, the internal reaction temperature has reached 129 °C when the shorting stops, which is ~50 °C higher than the threshold safety temperature and close to the melting temperature of the separator (~135 °C for polyethylene based separator)^31^,^32^, suggesting possible damage to the cell. Although the internal reaction temperature begins decreasing immediately after termination of shorting, it stays above threshold temperature of 80 °C for over 150 s. The surface temperature keeps increasing until reaching maximum value of 87.6 °C at 38.5 s, more than 20 s later than shorting termination. In comparison, the internal reaction temperature reaches maximum value at essentially the same time as shorting is terminated. The maximum surface temperature underestimates the maximum temperature inside the cell by more than 40 °C. Comparison of the internal reaction temperature and surface temperature shows again that the former responds much faster than the latter and provides a much more accurate monitor of highest temperature inside Li-ion battery cells under extreme conditions.

### Comparison of performance after shorting tests

**Figure 4:**
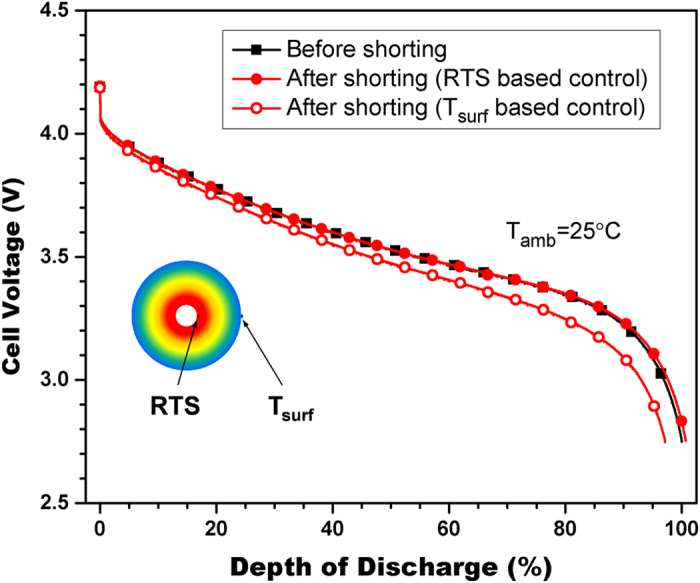
Performance of Li-ion battery cell after shorting tests with RTS-based control and surface temperature- based control. There is no performance decrease after shorting with RTS-based control while obvious decrease is observed after shorting with surface temperature- based control.

The results above show that cell temperature reaches 129 °C and stays higher than 80 °C for over 150 seconds with surface temperature- based control. Therefore, damage could have been caused to the Li-ion battery cell. To verify if the shorting cause damage and whether RTS-based control can avoid such damage, the Li-ion cell is tested after each shorting test. It is firstly fully charged and then fully discharged at 1C (1.6 A). The results are shown in Fig. 4. The performance before shorting test is also presented for comparison. It can be seen that there is no decrease of performance after shorting with RTS-based control. In comparison, cell performance shows obvious decrease (3%) after shorting with surface temperature- based control. The results confirm that RTS-based control can indeed avoid overheating and consequent damages.

## Discussion

The setting of termination temperature at 80 °C in above tests is conservative. When the termination temperature is set at higher temperature, much more serious damage could ensue with surface temperature- based control. We tested this hypothesis by further setting the termination temperature at 100 °C (See Supplement Figs 1, 2, 3). The highest internal temperature reached 137 °C and an additional 12% decrease of performance was observed after the shorting experiment with surface temperature- based control. In contrast, RTS-based control effectively detects abnormal temperature rise and prevents damages from overheating in the case of termination temperature at 100 °C.

Early detection and protection technologies are an essential part of large Li-ion batteries and their systems for vehicle, aircraft and grid energy storage applications. In this work we have developed a technique based on internal reaction temperature sensing (RTS) to detect abnormal temperature rise early and intervene early so as to prevent battery failure and catastrophic accident. Using a self-built 18650 Li-ion cell with capacity of 1.6 Ah, we showed that RTS-based control can detect abnormal temperature rise during shorting ~3 times earlier than surface temperature- based control. This technique prevents overheating of the Li-ion cell by ~50 °C and avoids subsequent damages from that overheating. The experimental results show that RTS-based control holds great promise for early detection and prevention of Li-ion battery safety breach. Further research based on this technique, including its application in other safety failure scenarios and in larger Li-ion batteries and packs, as well as development of more sophisticated sensors and control algorithms, is warranted.

Moreover, when cells with higher energy density experience shorting, internal temperature would be higher and much more serious damage could occur. Great challenges lie ahead to ensure safety of high energy density batteries, such as Li-S and Li-O_2_ batteries^33^,^34^, which are required for future electric transportation with much improved cruise range. The results in this study show that the RTS-based control will be an effective strategy for early detection of abnormal thermal behaviors and prevention of safety failures of future high energy density batteries.

## Methods

### Design and fabrication of Li-ion cells with RTS

As schematically shown in Fig. 1(a), cylindrical Li-ion battery cells (format 18650, diameter of 18 mm and height of 65 mm) are designed with embedded micro reaction temperature sensor (RTS) for internal reaction temperature diagnosis. The micro temperature sensor is located at innermost end of the reaction area of a cylindrical Li-ion cell where temperature is highest along the radial direction^21^. Comparing with fabricating conventional cylindrical Li-ion cells^35^, there are four additional steps in fabricating cells with RTS: (1) Coating micro temperature sensor with parylene for anti-corrosion in operating Li-ion cell condition; (2) Embedding the sensor at the reaction interface between negative electrode and separator near the innermost end of jelly roll during winding process; (3) Inserting the jelly roll with embedded sensor in a stainless steel can with a pre-drilled micro hole on the wall for extension of senor out of the can; (4) Sealing the micro hole on the wall of the can with epoxy before filling electrolyte and crimping of the cell.

Micro temperature sensors used in this study are T type micro thermocouples (600T, RTD Company, USA) with wire diameter of 80 um and an additional 10 um insulation. The micro thermocouples have no insulation on the measuring tip as received. A 10 μm layer of parylene is coated on the measuring tip using a special parylene evaporator at Penn State Materials Research Institute Nanofabrication Lab. With insulation, thickness of the sensor measuring tip is 100 um, identical to that of the wire. Another micro temperature sensor is placed on the outer surface of the cell to monitor surface temperature (T_surf_) and compare with RTS.

Experimental cells are made in the Battery Manufacturing Lab at The Pennsylvania State University, using LiNi_1/3_Co_1/3_Mn_1/3_O_2_ (NCM) and graphite as positive and negative electrode materials, respectively. Thicknesses of the positive electrode and negative electrode are 150 um and 140 um respectively, including current collecting collector and coating on both sides. The positive current collector is aluminum foil of 15 um and negative current collector is copper foil of 10 um. The separator is Celgard® 2320 PP/PE/PP trilayer membrane with thickness of 20 um. The electrolyte is 1.2 M LiPF_6_ in EC:EMC:DMC (20:20:60 v%).

### Development of experimental system to trigger and terminate short circuit

To verify the effectiveness of the RTS, we develop an experimental system that can trigger and terminate short circuit of an experimental Li-ion cell. Figure 1(b) shows the experimental system schematically. A shunt resistor (0.15 mΩ, ±0.5%, OHMITE, USA) is used to measure shorting current of the cell. The total external shorting resistance is 10 mΩ, including all resistance outside the cell, which is measured by a low resistance meter (3560, Hioki, Japan). A temperature controller (CN8201, OMEGA Engineering, USA) and a contactor (LEV200, Tyco Electronics, USA) are used for starting and terminating short circuit. A multi-channel data acquisition unit (34970A, Agilent Technologies, USA) is used to record the internal reaction temperature, surface temperature, current and voltage of the cell during test every 0.5 s. A battery tester (BT2000, Arbin Instruments, USA) is used to fully charge the cell before shorting test and to characterize cell performance after shorting test. The shorting test is carried out in a safety chamber which provides natural convection cooling condition.

### Cell charging protocol

The cell is fully charged using Constant Current-Constant Voltage (CC-CV) protocol (0.8 A, 4.2 V max, 0.032 A cut-off) at room temperature (25 ± 1 °C). Then the cell is rested for at least one hour, allowing open circuit voltage (OCV) and cell temperature to reach equilibrium, before shorting test or performance characterization after shorting test.

## Additional Information

**How to cite this article**: Zhang, G. *et al.* Reaction temperature sensing (RTS)-based control for Li-ion battery safety. *Sci. Rep.* **5**, 18237; doi: 10.1038/srep18237 (2015).

## Supplementary Material

Supplementary Information
