# Supplementary material for: Reaction temperature sensing (RTS)-based control for Li-ion battery safety
Source: Sci Rep. 2015 Dec 11;5:18237. doi: 10.1038/srep18237 (PMC4675999; doi:10.1038/srep18237)
Supplement: Supplementary Information [file srep18237-s1.doc]

**Supplementary Information**

Title: Reaction temperature sensing (RTS)-based control for Li-ion battery safety

Authors: Guangsheng Zhang, Lei Cao, Shanhai Ge, Chao-Yang Wang, Christian E. Shaffer, Christopher D. Rahn


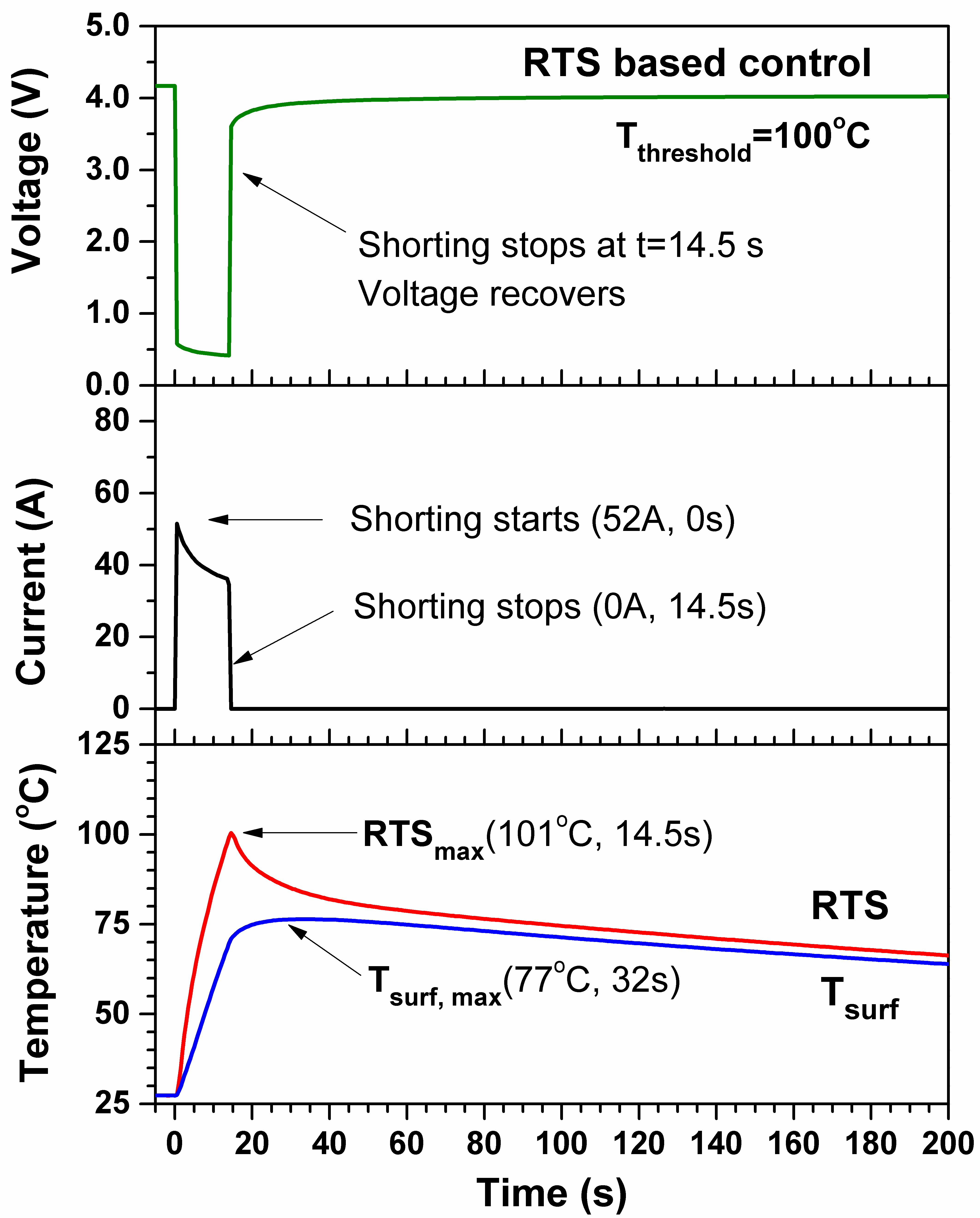


Supplementary Figure 1. Variation of cell voltage, current, reaction temperature (RTS) and surface temperature (T_surf_) of experimental Li-ion cell during shorting test with RTS-based control (T_threshhold_=100^o^C). The shorting is terminated successfully at 14.5 s when reaction temperature reaches 100^o^C.


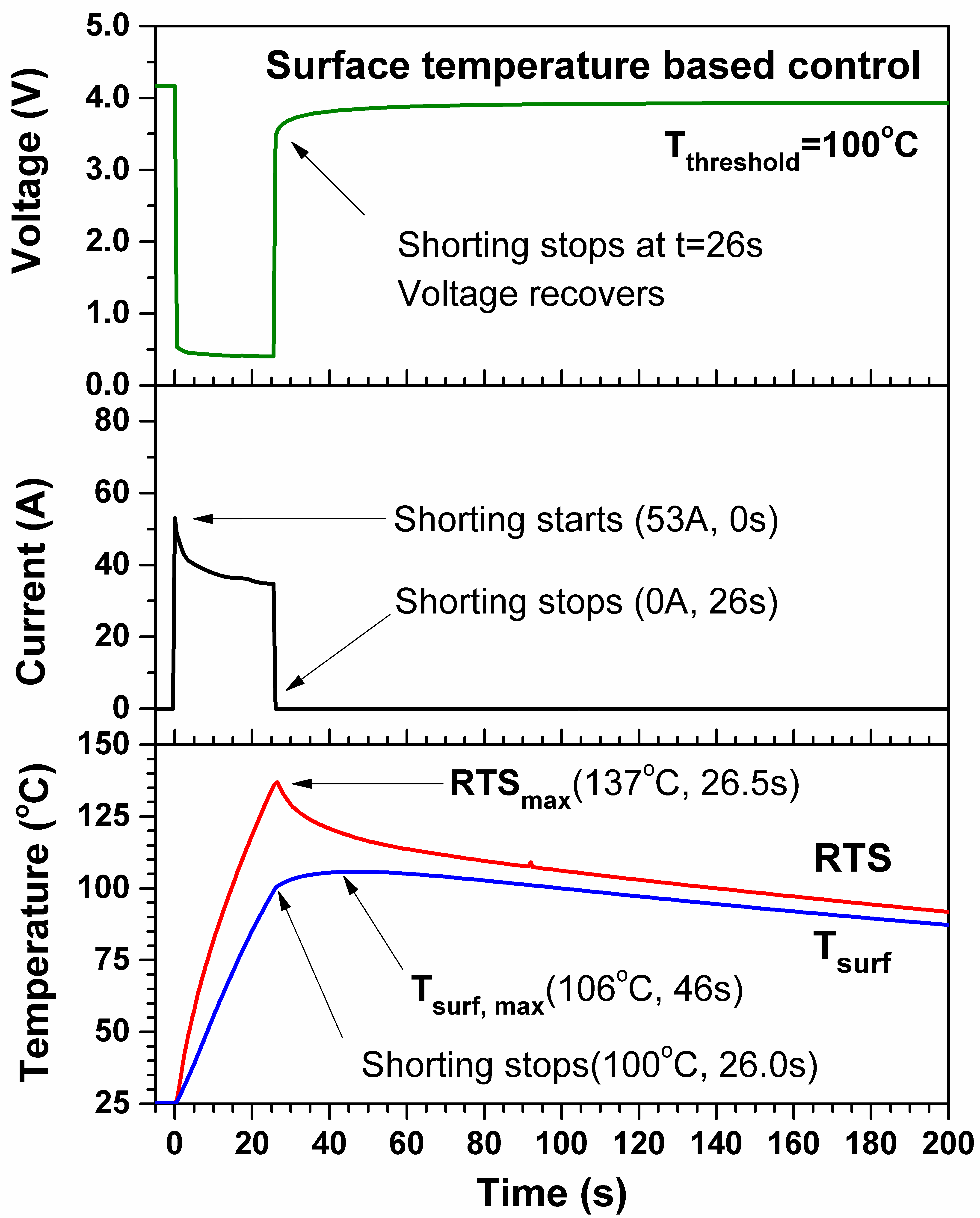


Supplementary Figure 2. Variation of cell voltage, current, reaction temperature (RTS) and surface temperature (T_surf_) of experimental Li-ion cell during shorting test with surface temperature- based control (T_threshhold_=100^o^C). The shorting is terminated successfully but reaction temperature reaches 137^o^C and it takes 22 seconds longer to terminate the shorting than that with RTS-based control.


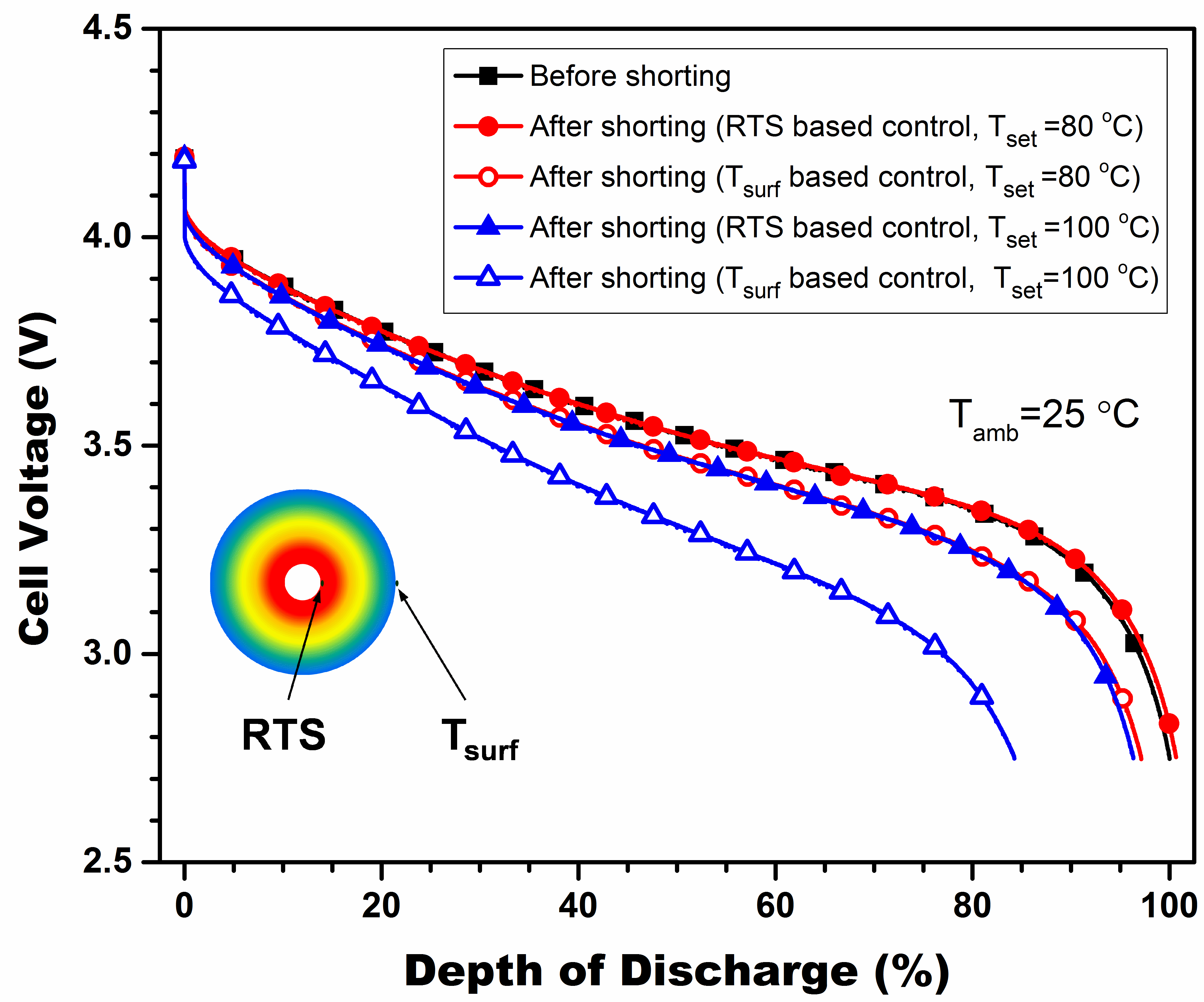


**Supplementary Figure 3.** Comparison of cell discharge performance before and after shorting tests. Note that the same cell is short-circuited 4 times according to the following order: (1) Short circuited and terminated with RTS-based control with T_threshhold_=80^o^C; (2) Short circuited and terminated with surface temperature-based control with T_threshhold_=80^o^C; (3) Short circuited and terminated with RTS-based control with T_threshhold_=100^o^C; (4) Short circuited and terminated with surface temperature-based control with T_threshhold_=100^o^C. There is no performance decrease after shorting test with RTS-based control but obvious performance decrease after shorting tests with surface temperature- based control.
